# Myopic shift in female mice after ovariectomy

**DOI:** 10.1038/s41598-024-74337-0

**Published:** 2024-10-03

**Authors:** Yan Zhang, Kiwako Mori, Heonuk Jeong, Junhan Chen, Yifan Liang, Kazuno Negishi, Kazuo Tsubota, Toshihide Kurihara

**Affiliations:** 1 Laboratory of Photobiology, Keio University School of Medicine, 35 Shinanomachi, Shinjuku-ku, Tokyo, 160- 8582 Japan; 2 Department of Ophthalmology, Keio University School of Medicine, 35 Shinanomachi, Shinjuku-ku, Tokyo, 160- 8582 Japan; 3 Tsubota Laboratory, Inc., 34 Shinanomachi, 304 Toshin Shinanomachi Ekimae Building, Shinju-ku, Tokyo, 160- 0016 Japan

**Keywords:** Myopia, Ovariectomy, Refraction, Axial length, Anterior chamber depth, Disease prevention, Eye diseases, Reproductive biology

## Abstract

Myopia is a global public health concern, with a higher prevalence in women than that in male. As the relationship between sex hormone and myopia remains unclear, we aimed to reveal the relationship between sex hormone and myopia by removing the ovaries of mice and measuring changes in ocular parameters related to myopia. Lens-induced myopia (LIM) surgeries were performed on 3-week-old male mice and age-matched female mice to evaluate the effects of sex on myopia development, which indicated a high degree of myopia and rapid progression of axial elongation in male mice. Bilateral ovariectomy (OVX) performed on 4-week-old female mice induced myopic refraction status in ovariectomized mice. Although axial length elongation was larger in ovariectomized mice than that in sham control mice, the result was insignificant. To further reveal the relationship between female sex hormones and myopia, LIM combined with OVX was performed, which revealed a magnified myopic refraction status in ovariectomized mice. Nevertheless, elongation of the anterior chamber depth in the − 30 D lens-treated eyes significantly increased in LIM + OVX mice compared with the frame-treated eyes in LIM + OVX mice and − 30 D lens-treated eyes in LIM + Sham surgery mice. Sex hormones play a role in regulating myopia development in female mice.

**Supplementary Information:**

The online version contains supplementary material available at 10.1038/s41598-024-74337-0.

## Introduction

Myopia, a common refractive error in which distant objects appear blurry while close objects are clear, is a global public health concern caused by a complex interaction between genetic predisposition and environmental exposures^1^. In most cases, myopia can be corrected with glasses, contact lenses, or refractive surgery; however, high myopia, which is defined as a refractive error of -6 diopters or worse, can lead to a range of complications that may result in vision loss or blindness^2^. At birth, the eye size and shape are determined by genetic factors, and the refractive state of the eye is typically hyperopic. During the early stages of development, the eye is highly sensitive to environmental factors, such as visual experience, lighting conditions, and nutritional status, which can affect the growth and maturation of eye structures. As the eye grows, the cornea and lens begin to change shape, which affects their refractive power. By the age of 6, most children have achieved emmetropia, implying that the refractive powers of the cornea and lens perfectly match the length of the eye, resulting in clear vision at a distance without the need for corrective lenses^3^. Therefore, several studies have indicated that hyperopia is commonly observed in preschoolers aged 3–6 years^4–7^. However, some children continue to experience refractive changes during adolescence and the risk of developing myopia increases during this stage. The prevalence of myopia has been reported to increase from school ages (6–19 years)^8^ and is expected to affect approximately 50% of people worldwide by 2050^9^.

The prevalence of myopia varies with age in different genders. Approximately 10% ^4,7,10–12^ of children aged < 6 years is myopia, we and other researchers have found that male sex is significantly associated with myopia^6^ or premyopia^4^, and boys have a longer axial length (AL)^6,7^ than girls at preschool age. Nevertheless, a significant number of studies have shown that girls are more likely to develop myopia earlier and have a higher prevalence of myopia than boys after reaching school age^13–16^. This shift in myopia prevalence between genders can be attributed to the fact that girls tend to experience puberty at an earlier age. And it is widely accepted that the earlier the age of myopia onset, the greater the possibility of developing high myopia^17,18^. Females continue to have a higher prevalence of myopia and high myopia not only in early adulthood^19–21^ but also in those aged ≥ 40 years^22^. In addition, females have faster myopic progression^19^ and a higher prevalence of ocular comorbidities, such as myopic macular pathology^23^ and myopic neovascularization, than males^19^. Therefore, female sex is considered a risk factor for myopia by many researchers.

A nationwide cross-sectional study conducted in China indicated that girls’ earlier puberty contributes significantly to their higher prevalence of myopia^24^, and a Korean research team also found that women with a younger menarche age have a higher risk of developing severe myopia^25^. They suspected that an increase in female sex hormones, such as oestrogen, or growth spurts during puberty could be associated with the change of refractive status. Oestrogen produced by the ovary is an important female hormone, and its receptors have been detected everywhere in the eye, including the cornea, lens, iris, ciliary body, retina, lacrimal gland, meibomian gland, and conjunctiva^26^. Numerous studies have revealed that oestrogen levels play a role in ocular surface homeostasis^27^ and exert protective effects against some retinal disorders, such as age-related macular degeneration and diabetic retinopathy^28^, while the relationship between female sex hormones and myopia remains unknown.

Ovariectomy (OVX) is a surgical procedure that involves the removal of ovaries from female animals to study the effects of ovarian removal and the subsequent reduction in circulating sex hormones, particularly oestrogen, on various physiological and pathological processes. It has been used to study various conditions, including osteoporosis^29^, cardiometabolic disorders^30^, and inflammatory diseases^31^. In the field of ophthalmology research, the procedure is commonly used in studies on the relationship between oestrogen and dry eye^32^, whereas the relationship between sex hormones and myopia is yet to be established. In this study, the relationship between female sex hormones and myopia was investigated by removing the ovaries of mice and measuring changes in myopia-related ocular parameters.

## Results

### Sex-related differences in myopia development and ocular parameters following lens-induced myopia (LIM) in young mice

**Fig. 1:**
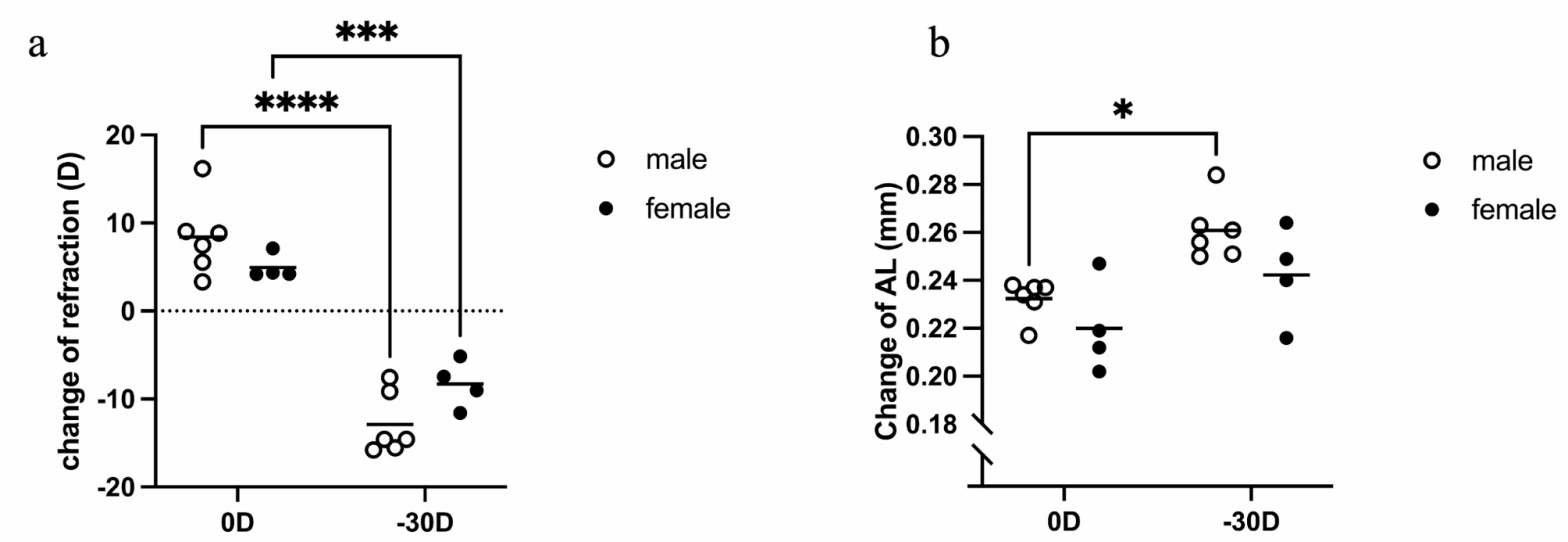
Sex-related differences in myopia development and ocular parameters following LIM in young mice. (**a**) Both male and female mice revealed a high degree of myopia in -30 D lens eyes, but only male mice showed significant myopic shift. (**b**) Changes in AL was significantly more in -30 D lens eyes in male mice than those in -30 D lens eyes in female mice, while changes in AL in female mice were not significant. Male mice: *n* = 6, female mice: *n* = 4. *P < 0.05, ***P < 0.001, ****P < 0.0001, two-way ANOVA tests.

LIM was performed on 3-week-old male and age-matched female mice to investigate the effects of sex on myopia development. Ocular parameters, such as refraction status and AL, were measured immediately before and 3 weeks after LIM surgeries. Unlike the significant changes in refraction and AL in male mice, female mice exhibited a significant myopic shift in refractive status but did not display notable changes in AL. Male mice showed a propensity of a higher degree of myopia and a faster progression of axial elongation than female mice, these differences did not reach statistical significance (Fig. 1a,b).

### Mice that underwent bilateral OVX showed myopic shift

**Fig. 2:**
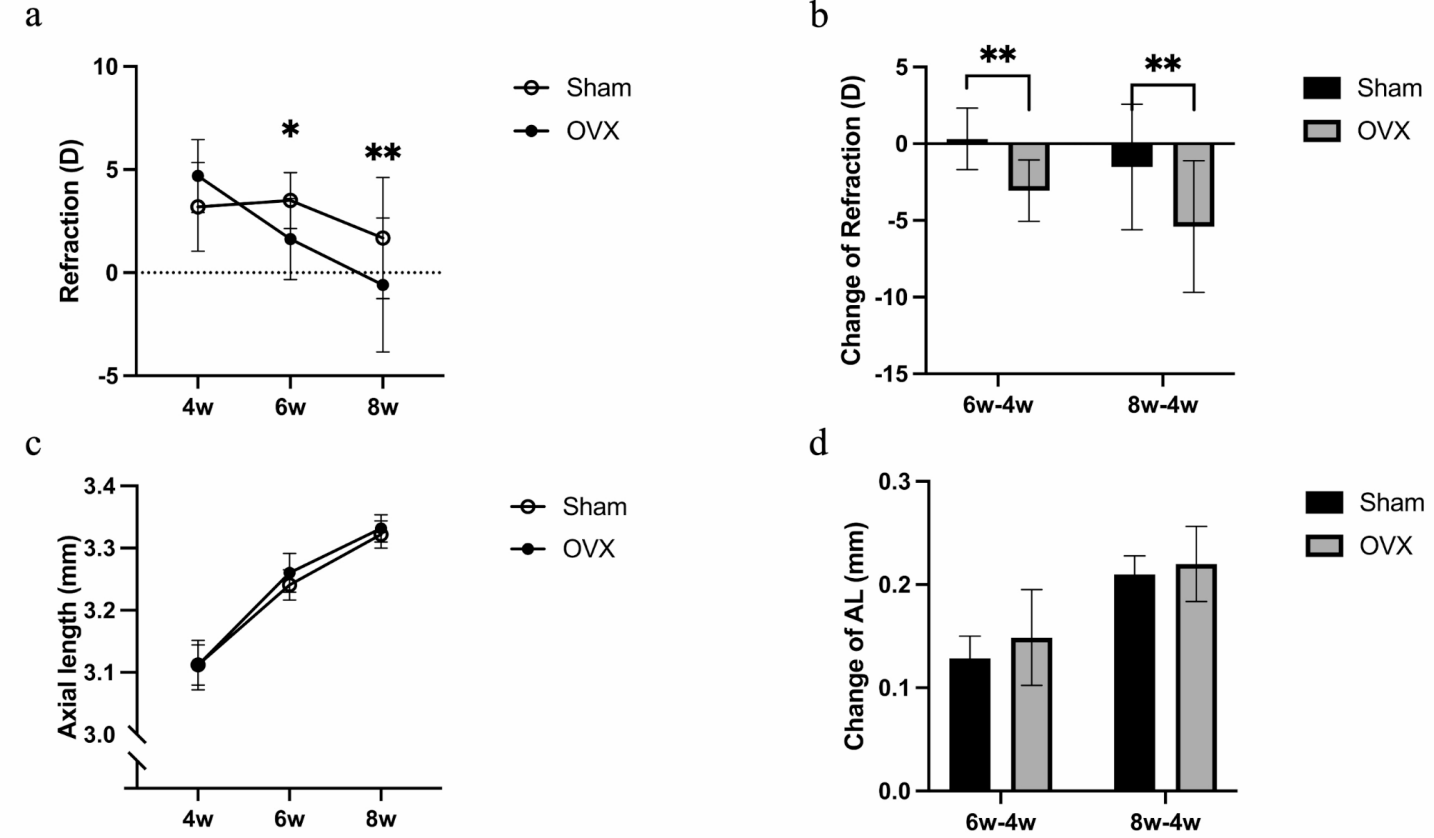
Female mice showed myopic shift after ovariectomy. (**a**) The refraction status of mice that underwent ovariectomy became myopic compared with mice that underwent only sham surgery (control group) when 6- (2 weeks after surgery) and 8- (4 weeks after surgery) week-old. (**b**) Change in refraction status 2 and 4 weeks after surgery indicated significant difference between ovariectomy and sham control group. (**c**) The AL did not show significant difference after surgery. (**d**) Changes in AL did not show significant difference after surgery. *n* = 5. *P < 0.05, **P < 0.01, multiple unpaired t tests. Error bars indicate mean ± SD.

**Fig. 3:**
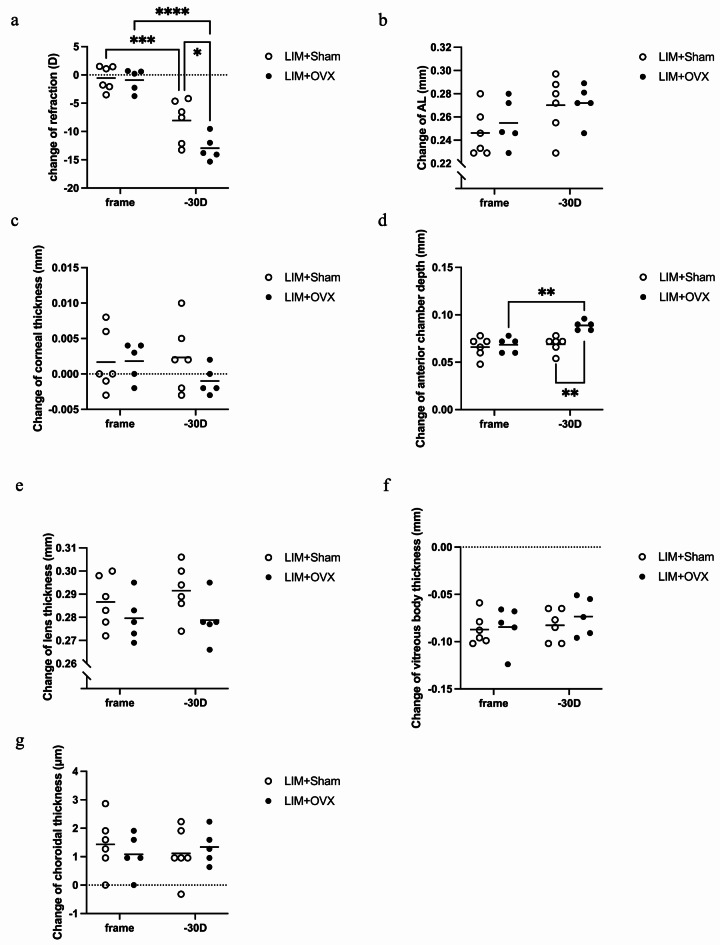
Mice that underwent ovariectomy + LIM showed more myopic refraction status and deeper anterior chamber than LIM without ovariectomy. (**a**) Both sham control and ovariectomized mice indicated myopia in -30D lens eyes, and ovariectomized mice showed more myopic shift than the sham control mice. (**b**,**c**,**e**,**f**,**g**) No significant difference was found in the AL, corneal thickness, lens thickness, vitreous body thickness, and choroidal thickness of frame-treated eyes or the − 30 D lens-treated eyes, and no difference was found in the sham control and ovariectomy groups. (**d**) ACD was deeper in ovariectomized mice than that in the sham control mice and deeper in -30 D lens-treated eyes than the frame-treated eyes of ovariectomized mice. *n* = 6 in control group, *n* = 5 in OVX group. *P < 0.05, **P < 0.01, ***P < 0.001, ****P < 0.0001, two-way ANOVA tests.

In the experiment of mice undergoing OVX only, the refraction of the OVX shifted towards myopia 2 and 4 weeks after OVX (Fig. 2a,b). The AL and change in AL in the OVX group showed a tendency of elongation, but the results were not significantly different (Fig. 2c,d). In the experiment with mice that underwent LIM + OVX, eyes treated with − 30 D lenses showed a significantly larger refractive change than those treated only by wearing frames in both the control and surgery groups (Fig. 3a). Moreover, the refractive statuses of the-30 D lens-treated eyes in the OVX group were more myopic than those in the control group (Fig. 3a). The change in AL in both the control and LIM + OVX groups revealed a slight increase in the − 30 D lens-treated eyes, and the LIM + OVX group showed a larger change in AL in both eyes than the control group; however, the results were not significantly different (Fig. 3b). The change in corneal thickness was small in the − 30 D lens-treated eyes in the LIM + OVX group (Fig. 3c), and the change in lens thickness (LT) was small in both eyes of the LIM + OVX mice (Fig. 3e); however, no significant differences were found. The anterior chamber depth (ACD) was significantly deeper in the − 30 D lens-treated eyes in the LIM + OVX group than that in the − 30 D lens-treated eyes in the control group and was deeper in the − 30 D lens-treated eyes than that in the frame-treated eyes of LIM + OVX mice (Fig. 3d). No significant differences were observed in the changes in vitreous body depth or choroidal thickness between the two groups (Fig. 3f,g).

## Discussion

We measured changes in ocular parameters, such as refraction, AL, ACD, LT, vitreous body depth, and choroidal thickness, after removing bilateral ovaries from 4-week-old female mice to investigate the effect of female sex hormones on the eyes. We found that bilateral ovariectomy induced myopia, and this effect was magnified on combining LIM with OVX. In the LIM + OVX experiment, the − 30 D lens-treated eyes showed more myopia than the frame-treated eyes in OVX group; furthermore, -30 D lens-treated eyes showed more myopia in the OVX group than those in the control group. AL elongation was greater in the OVX group than that in the control group; however, the difference was not statistically significant. Nevertheless, ACD elongation in the − 30 D lens-treated eyes significantly increased in the OVX group compared to that in the frame-treated eyes in the OVX group and − 30 D lens-treated eyes in the control group. In addition, male mice showed a higher degree of myopia and a more rapid progression of axial elongation than female mice after LIM.

Studies have shown that females have a higher prevalence of myopia than males^13–16,19–21,33,34^, and an earlier onset of puberty in females may be associated with the onset of myopia^24,25,35^. Growth spurts and puberty are related; however, distinct processes occur during human development, and it is difficult to distinguish the effects of growth spurts or puberty on myopia. Clinical studies have shown that GnRH therapy slows myopic progression in girls with central precocious puberty^36^, but GnRH therapy also suppresses both sex and growth hormones, making it difficult to distinguish their individual effects. Another clinical study found that growth hormone replacement therapy in children with congenital growth hormone deficiency caused significant AL elongation and a myopic shift in refraction^37^. A longitudinal study from Singapore suggested that rather than pubertal changes, growth spurts and possible growth hormone surges may be significant mediators of myopia progression^38^.

Previous studies have shown that female sex hormones, especially oestrogen, play important roles in the cardiovascular system protection^39^ and neuroprotection^40^. Ophthalmology studies have revealed that oestrogen plays a significant role in the stability of ocular surface^27,32^. The menstrual cycle^41^, pregnancy^42^, and menopause^43^ that cause changes in oestrogen level affects corneal thickness, corneal curvature, and ocular surface stability. Additionally, oestrogen protects against other ocular conditions, such as cataract^35^, primary open angle glaucoma^44^, posterior vitreous detachment^45^, age-related macular degeneration, and diabetic retinopathy^28^. However, the association between sex hormone levels and myopia remains unclear. A previous study found a high level of testosterone in patients with high myopia, and oestrogen levels significantly increased and progestogen levels significantly decreased in male patients with myopia^46^. The study, therefore, claimed that sex hormone levels were associated with myopia, whereas another study found no statistically significant difference in serum sex hormone levels between patients with high myopia and those with emmetropia^47^. Our research found that male mice showed a higher degree of myopia and more rapid progression of axial elongation than female mice after LIM and removing bilateral ovaries of mice led to myopic shift in refraction statuses and deep anterior chamber. The cornea tended to become thinner and the AL tended to increase in ovariectomized female mice, but the difference was not significant. These changes in ocular parameters were consistent with human data, in which ACD was negatively correlated with corneal power and positively correlated with AL^48^. This suggests that sex hormones play a role in regulating myopia development in female mice.

Oestrogen plays a significant role in regulating various cellular processes, including the extracellular matrix (ECM) composition^49^, actin cytoskeleton organization^50,51^, and mechanotransduction pathways^52^, which are crucial for maintaining the structural integrity and function of ocular tissues. Additionally, oestrogen is known to influence nitric oxide production and vascular endothelial growth factor (VEGF) expression^53,54^, which has vasodilatory effects^55,56^ and angiogenesis effects on choroidal vessels, potentially affecting blood flow and ocular health. Choroidal thickness has received extensive attention as a clinical biomarker in myopic research^57^. It is generally believed that choroidal thickness is negatively correlated with myopia severity^58^. According to our previous study, VEGF is essential for the development and maintenance of choroidal vessels^59^. Therefore, the removal of both ovaries is expected to cause choroidal thinning. However, our results showed no significant changes in choroidal thickness, and we believe that myopia induced by bilateral OVX mainly originated from changes in the anterior segment of the eye.

In contrast to the growth hormone surges possibly related to the onset of myopia, the removal of bilateral ovaries in mice causes a decrease in female sex hormones, which, in turn, causes the onset of myopia. Female sex hormones may have a potential role in regulating myopia; changes in female sex hormones caused by the menstrual cycle, pregnancy, and menopause, and their effects on myopia onset and progression require further study to fully understand the mechanisms underlying myopia development and female sex hormones and to explore potential therapeutic strategies targeting these mechanisms.

## Methods

### Mice

All procedures were approved by the Ethics Committee on Animal Research of the Keio University School of Medicine adhered to the Association for Research in Vision and Ophthalmology Statement for the Use of Animals in Ophthalmic and Vision Research, the Institutional Guidelines on Animal Experimentation at Keio University (Approval number: A2022-242), and the Animal Research: Reporting of In Vivo Experimental (ARRIVE) guidelines for the use of animals in research. Our study was also conducted in accordance with Sex and Gender Equity in Research guidelines. C57BL6/J mice (CLEA Japan, Tokyo, Japan) were raised in standard transparent mouse cages (29 × 18 × 13 cm) in an air-conditioned room kept at 23 ± 3 ℃ with a 12-h dark/light cycle and free access to a standard diet (CE-2, CLEA Japan, Tokyo, Japan) and tap water. Each cage housed no more than five mice.

### Ocular parameters

 Ocular parameters were measured as mentioned in previous studies^59–61^. In short, an infrared photorefractor (Steinbeis Transfer Center, Tübingen, Germany) was used to determine the refractive state. Tropicamide and phenylephrine hydrochloride solutions (Mydrin-Pophthalmic solution, Santen Pharmaceutical) were administered to the mouse eye 5 min before measurement to ensure mydriasis and cycloplegia. A combination of midazolam (Sandoz K.K.), medetomidine (Domitor; Orion Corporation), butorphanol tartrate (Meiji Seika Pharma Co., Ltd.) was used to induce general anaesthesia. Refraction was measured along the optical axis. Following refraction measurements, AL, corneal thickness, ACD, lens thickness, vitreous body depth, and choroidal thickness were measured using a spectral domain- optical coherence tomography (OCT) system (Envisu R4310, Leica)^59,62^. AL is the distance between the vertex of the cornea and RPE layer near the optic nerve. An OCT system was used to measure choroidal thickness according to previous studies. ImageJ software (NIH) was used to calculate the area of the circumference (0.5 mm) from the disk circled at the border of the RPE and the posterior surface of the choroid. The average choroidal thickness was calculated by dividing the circumference by the area.

### LIM model

LIM was performed on mice that underwent LIM + OVX surgery. According to previous reports^63^, MMB was administered under general anaesthesia to 3-week-old mice. The scalp was dissected to expose a 0.8 cm^2^ area of the skull, and the periosteum was removed with etching fluid. Subsequently, a pair of eyeglasses was adhered to the mouse head using a self-curing dental adhesive system (Super-Bond, SUN MEDICAL). The eyeglasses were specially designed for the mice using a three-dimensional printer. The eyeglasses had a joint that allowed the left and right frame positions to be adjusted to fit a mouse skull or removed for cleaning. A Japanese manufacturer made eyeglass lenses specifically for mice using human hard contact lenses. As an internal control, all left sides of the eyeglasses used in this study had frames only, whereas the right side had − 30 D lenses attached. The glass was removed from each mouse at least twice a week for cleaning.

### Ovariectomy

 An intraperitoneal injection of general anaesthetic agent of MMB (a mixture of medetomidine, midazolam and butorphanol) was used to anesthetize the 4-week-old mice. The ovaries were identified and removed from the adherent tissue following an incision in the lower abdomen. Mice subjected to the same manipulations, except for ovary removal, served as sham controls and were labelled as ‘control’ animals. All the surgical procedures were performed by the same surgeon. The success of OVX surrey was validated on established literature^64^, which consistently shows significant increase of body weight (Supplementary Fig. 1) following OVX.

### Statistical analysis

 All data are presented as the means ± standard deviation and were analysed using GraphPad Prism 9.0. Multiple t-tests were used to compare the mean variables of the two groups at different time points. Intergroup analyses were performed using two-way analysis of variance (ANOVA), followed by the Tukey–Kramer multiple-comparison test. The Tukey-Kramer post-hoc analysis was chosen to account for the unequal sample sizes across groups, providing a more conservative and accurate assessment of the differences between groups. Statistical significance was set at *P* < 0.05.

## Electronic supplementary material

Below is the link to the electronic supplementary material.

Supplementary Material 1

Supplementary Material 2

## Data Availability

All data generated or analysed during this study are included in this published article and its supplementary information file.

## Abbreviations

LIM: Lens-induced myopia
OVX: Ovariectomy
AL: Axial length
LT: Lens thickness
ACD: Anterior chamber depth
VEGF: Vascular endothelial growth factor
