# Supplementary material for: Myopic shift in female mice after ovariectomy
Source: Sci Rep. 2024 Oct 3;14:22946. doi: 10.1038/s41598-024-74337-0 (PMC11449924; doi:10.1038/s41598-024-74337-0)
Supplement: Supplementary file 2 — Supplementary Material 2 [file 41598_2024_74337_MOESM2_ESM.docx]

**Supplementary Information for**

Myopic Shift in Female Mice after Ovariectomy

Yan Zhang, Kiwako Mori, Heonuk Jeong, Junhan Chen, Yifan Liang, Kazuno Negishi, Kazuo Tsubota, Toshihide Kurihara

Toshihide Kurihara, MD, PhD

Laboratory of Photobiology, Department of Ophthalmology, Keio University School of Medicine; 35 Shinanomachi, Shinjuku-ku, Tokyo 160-8582, Japan.

Tel: +81-3-5363-3204, Fax: +81-3- 5363-3274

E-mail: [kurihara@z8.keio.jp](mailto:kurihara@z8.keio.jp)

Kazuo Tsubota, MD, PhD

Tsubota Laboratory, Inc., 34 Shinanomachi, 304 Toshin Shinanomachi Ekimae Building, Shinju-ku, Tokyo 160-0016, Japan.

Tel: +81-3-6384-2866

E-mail: [tsubota@tsubota-lab.com](mailto:tsubota@tsubota-lab.com)

**This file includes:**

**Figures S1**

**References**


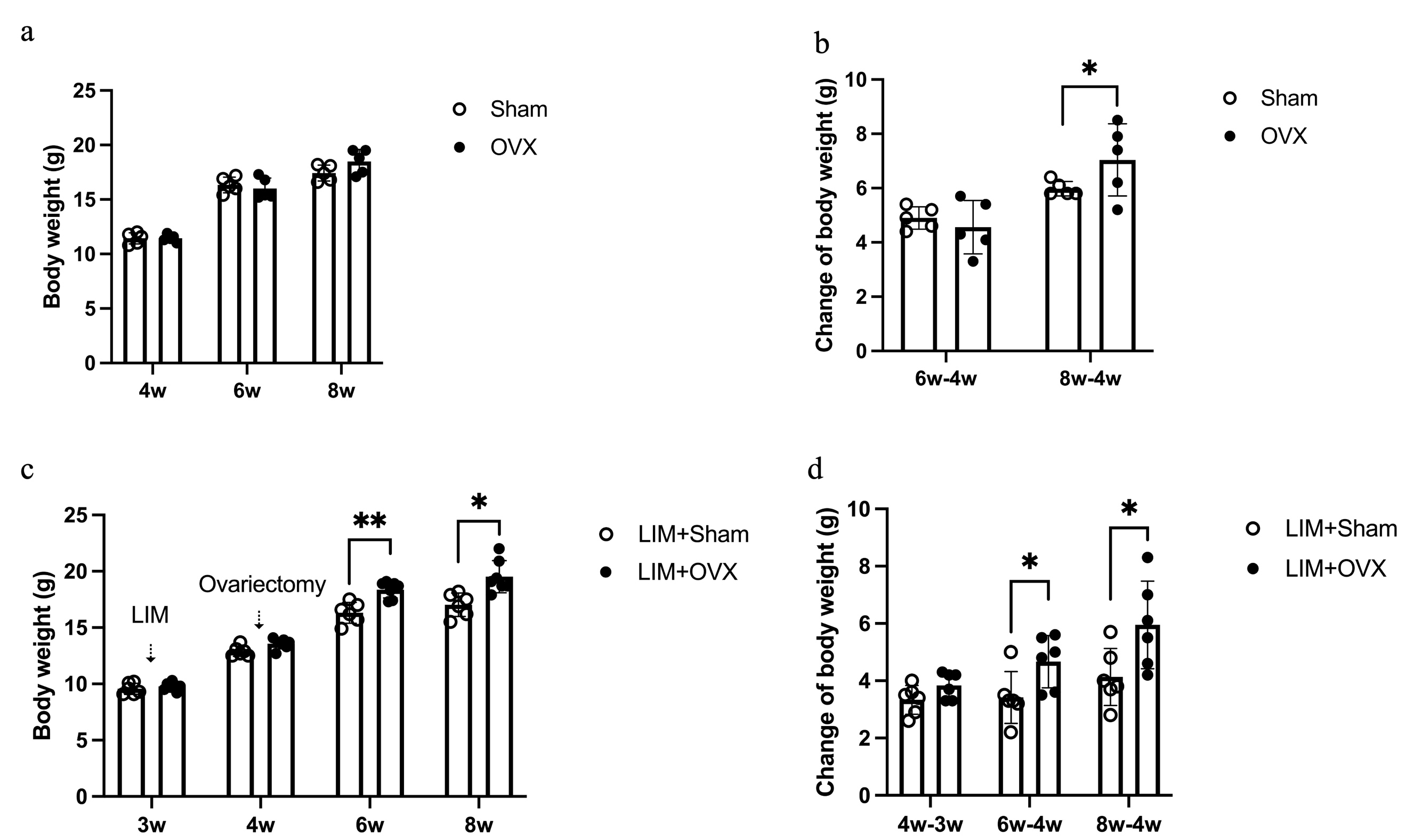


**Fig. S1.** **Bilateral OVX Resulted in Body Weight Gain.** A significant increase in body weight was observed after OVX, as previously reported^1,2^. In the OVX only group, no significant differences were found in body weight between the control and OVX groups at baseline and 2 and 4 weeks after surgery **(a)**, while compared to the baseline body weight, the change in body weight 4 weeks after surgery increased in the OVX mice **(b)**. In the experiment of mice undergoing LIM when 3 weeks old and OVX when 4 weeks old, the body weight showed no significant differences at baseline and 1 week after LIM **(c)**, and the change in body weight at 1 week after LIM indicated no significant difference either **(d)**. Although the body weight at 2 and 4 weeks after OVX increased significantly compared to the body weight before OVX (4 weeks old), body weight also increased in OVX mice, indicating that body weight only increased after OVX but not after LIM. n=6. *P < 0.05, **P < 0.01, multiple unpaired t tests. Error bars indicate mean ± SD.

**References**

1. Davidge, S. T., Zhang, Y. & Stewart, K. G. A comparison of ovariectomy models for estrogen studies. Am J Physiol Regul Integr Comp Physiol 280, 904–907 (2001).

2. Shimomura, K. et al. Is leptin a key factor which develops obesity by ovariectomy? Endocrine Journal vol. 49 417–423 Preprint at https://doi.org/10.1507/endocrj.49.417 (2002).
